# A Novel Integrative Mechanism in Anxiolytic Behavior Induced by Galanin 2/Neuropeptide Y Y1 Receptor Interactions on Medial Paracapsular Intercalated Amygdala in Rats

**DOI:** 10.3389/fncel.2018.00119

**Published:** 2018-05-01

**Authors:** Manuel Narváez, Dasiel O. Borroto-Escuela, Luis Santín, Carmelo Millón, Belén Gago, Antonio Flores-Burgess, Miguel A. Barbancho, Miguel Pérez de la Mora, José Narváez, Zaida Díaz-Cabiale, Kjell Fuxe

**Affiliations:** ^1^Instituto de Investigación Biomédica de Málaga, Facultad de Medicina, Universidad de Málaga, Málaga, Spain; ^2^Department of Neuroscience, Karolinska Institute, Stockholm, Sweden; ^3^Department of Biomolecular Science, Section of Physiology, University of Urbino, Urbino, Italy; ^4^Grupo Bohío-Estudio, Observatorio Cubano de Neurociencias, Yaguajay, Cuba; ^5^Instituto de Investigación Biomédica de Málaga, Facultad de Psicología, Universidad de Málaga, Málaga, Spain; ^6^Instituto de Fisiología Celular, Universidad Nacional Autónoma de México, Mexico City, Mexico

**Keywords:** galanin receptor 2, neuropeptide Y Y1 receptor, interaction, heteroreceptors complexes, amygdala, anxiety

## Abstract

Anxiety is evoked by a threatening situation and display adaptive or defensive behaviors, found similarly in animals and humans. Neuropeptide Y (NPY) Y1 receptor (NPYY1R) and Galanin (GAL) receptor 2 (GALR2) interact in several regions of the limbic system, including the amygdala. In a previous study, GALR2 enhanced NPYY1R mediated anxiolytic actions on spatiotemporal parameters in the open field and elevated plus maze, involving the formation of GALR2/NPYY1R heteroreceptor complexes in the amygdala. Moreover, the inclusion of complementary ethological parameters provides a more comprehensive profile on the anxiolytic effects of a treatment. The purpose of the current study is to evaluate the anxiolytic effects and circuit activity modifications caused by coactivation of GALR2 and NPYY1R. Ethological measurements were performed in the open field, the elevated plus-maze and the light-dark box, together with immediate early gene expression analysis within the amygdala-hypothalamus-periaqueductal gray (PAG) axis, as well as *in situ* proximity ligation assay (PLA) to demonstrate the formation of GALR2/NPYY1R heteroreceptor complexes. GALR2 and NPYY1R coactivation resulted in anxiolytic behaviors such as increased rearing and head-dipping, reduced stretch attend postures and freezing compared to single agonist or aCSF injection. Neuronal activity indicated by cFos expression was decreased in the dorsolateral paracapsular intercalated (ITCp-dl) subregion of the amygdala, ventromedial hypothalamic (VMH) nucleus and ventrolateral part of the periaqueductal gray (vlPAG), while increased in the perifornical nucleus of the hypothalamus (PFX) following coactivation of GALR2 and NPYY1R. Moreover, an increased density of GALR2/NPYY1R heteroreceptor complexes was explicitly observed in ITCp-dl, following GALR2 and NPYY1R coactivation. Besides, knockdown of GALR2 was found to reduce the density of complexes in ITCp-dl. Taken together, these results open up the possibility that the increased anxiolytic activity demonstrated upon coactivation of NPYY1R and GALR2 receptor was related to actions on the ITCp-dl. GALR2-NPYY1R heteroreceptor complexes may inhibit neuronal activity, by also modifying the neuronal networks of the hypothalamus and the PAG. These results indicate that GALR2/NPYY1R interactions in medial paracapsular intercalated amygdala can provide a novel integrative mechanism in anxiolytic behavior and the basis for the development of heterobivalent agonist drugs targeting GALR2/NPYY1R heteromers, especially in the ITCp-dl of the amygdala for the treatment of anxiety.

## Introduction

Anxiety is defined as a negative emotional state and defensive reaction characterized by a feeling of worries, apprehension, and uncertainty stemming from the anticipation of potential threats (Davis and Shi, 1999). In anxiety conditions, humans and animals face an ambiguous threat stimulus and experience a high level of uncertainty and unpredictability (Lowry et al., 2005). This threatening situation evokes an evolutionarily conserved brain state which triggers adaptive or defensive behaviors, ranging from risk assessment and freezing to flight and defensive attack to avoid or reduce potential harm (Blanchard et al., 2001; LeDoux, 2012; Anderson and Adolphs, 2014). In fact, human defensive behaviors to threat scenarios are analogous those seen in non-human mammals (Blanchard et al., 2001).

This fact allows using experimental rodents to study the neurobiological basis of anxiety and the screening for novel targets and anxiolytic compounds. In the open-field, the elevated plus-maze and the light-dark box, main tests used for unconditioned anxiety (Prut and Belzung, 2003; Carobrez and Bertoglio, 2005; Treit et al., 2010) mice and rats show a spontaneous natural preference for unlit and protected spaces, and seem to avoid the open and/or lit areas. This natural response is used as an indicator of anxiety in animals and is analyzed as conventional spatiotemporal indices. Moreover, complementary ethological parameters were introduced to study the effects of drugs in the assessment of anxiety, along with spatiotemporal parameters in animals (Rodgers and Dalvi, 1997; Carobrez and Bertoglio, 2005). In fact, ethological measurements such as rearing, stretch-attend postures (SAP) or freezing provide a more comprehensive profile on the anxiolytic effects of a treatment (Calatayud et al., 2004; Rey et al., 2012). Rearing behavior has been considered as an exploratory behavior, thus increased rearing seems to be associated with reduced anxiety (Camara et al., 2015). In addition, reduced freezing or SAP was observed with anxiolytic drug treatments (Rey et al., 2012).

A long-standing question in fear and anxiety research has been how brain circuits generate defensive behaviors, which have been involved in normal fear and maladaptive anxiety (Deisseroth, 2014; Tovote et al., 2015). This topic is particularly crucial for innate anxiety as most of the information gathered on anxiety circuits comes from experiments using conditioned models of anxiety. Thus, according to the most popular theory (LeDoux, 2007), relevant information from the environment follows a linear pathway reaching the amygdaloid basolateral nucleus where after being processed it is conveyed to the amygdaloid central nucleus where a proper anxiogenic response is implemented. Some evidence has however shown that anxiogenic information may flow in a more distributed way (Killcross et al., 1997) and that even many neuronal pathways have been involved in innate anxiety (Gross and Canteras, 2012). In this regard, it is essential that several brain regions have been implicated in innate anxiety, including the amygdala, medial hypothalamic zone and downstream midbrain periaqueductal gray (PAG) region. This amygdala-hypothalamus-PAG axis may constitute the executive system for anxiety since defensive behavioral repertoire of animals can be evoked along the whole trajectory of this system (Martinez et al., 2011; Gross and Canteras, 2012; LeDoux, 2012). In these regions, neuropeptides and their receptors have received particular attention as attractive therapeutic targets in emotional disorders, including anxiety (Holmes et al., 2003a; Kormos and Gaszner, 2013).

Among them, Neuropeptide Y (NPY) is a 36-amino acid peptide isolated from brain extracts (Tatemoto et al., 1982), and found to be one of the most abundant neuropeptides within the brain (Sajdyk et al., 2004). NPY has been suggested to be a key component in the stress response, showing anxiolytic properties (Rotzinger et al., 2010). Many reports indicate that the anxiolytic activity of NPY is primarily mediated by Y1 receptors (NPYY1R) (Karlsson et al., 2008; Lach and de Lima, 2013), affecting not only the spatiotemporal but also some ethological parameters in different behavioral tasks (Karlsson et al., 2005).

Galanin (GAL), is also a neuropeptide widely distributed in the central nervous system (Jacobowitz et al., 2004). The GAL role in anxiety behaviors depends on the route, site of its administration and also on the intensity of stress-conditions (Holmes et al., 2003b; Holmes and Picciotto, 2006). So, GAL anxiolytic-like effects might occur mainly under high-stress and silent otherwise (Karlsson et al., 2005). Through GAL receptors (GALR), this neuropeptide seems to participate in anxiety-like behaviors via modulation of neuroendocrine and monoaminergic systems (Wrenn and Holmes, 2006; Fuxe et al., 2012).

Several GALR and NPYY1R interactions were described in distinct regions of the limbic system (Diaz-Cabiale et al., 2011; Narvaez et al., 2015, 2016). In the amygdala, a facilitatory GALR/NPYY1R interaction was demonstrated involving the formation of GALR2/NPYY1R heteroreceptor complexes. Moreover, the activation of GALR2 enhanced NPYY1R-mediated anxiolytic actions on spatiotemporal parameters in the open field and elevated plus maze (Narvaez et al., 2015).

The purpose of the current study is to evaluate the anxiolytic effects and circuit activity modifications caused by coactivation of GALR2 and NPYY1R. Ethological measurements were performed in the open field, the elevated plus-maze and the light-dark box, together with immediate early gene expression analysis within the amygdala-hypothalamus-PAG axis, as well as *in situ* proximity ligation assay (PLA) to demonstrate the formation of GALR2/NPYY1R heteroreceptor complexes.

## Materials and Methods

### Animals

Male Sprague-Dawley rats from CRIFFA (Barcelona; 200–250 g; 6–8 weeks) had free access to food pellets and tap water. They were maintained under the standard 12 h dark/light cycle, with controlled temperature (22 ± 2°C) and relative humidity (55–60%). All procedures concerned with housing, maintenance, and experimental treatment of the rats were approved by the Local Animal Ethics, Care and Use Committee for University of Malaga, Spain. Guidelines for animal experiments were carried out in accordance with EU Directive 2010/63/EU and Spanish Directive (Real Decretory 53/2013) recommendations. Detailed description on animal intracerebral cannulations is available in Supplement Material.

### Drugs Used

Solutions were freshly prepared and the peptides were dissolved in artificial cerebrospinal fluid (aCSF, composition is (in mM) 120 NaCl, 20 NaH_2_CO_3_, 2 KCl, 0.5 KH_2_PO_4_, 1,2 CaCl_2_, 1,8 MgCl_2_, 0,5 Na_2_SO_4_, and 5,8 D-glucose, pH 7.4). Galanin (GAL), Galanin Receptor 2 (GALR2) Agonist M1145, NPYY1 Receptor (NPYY1R) Agonist [Leu^31^,Pro^34^]NPY and GALR2 Antagonist M871 were obtained from Tocris Bioscience (Bristol, United Kingdom). Detailed descriptions are available in Supplement Material on intracerebroventricular (icv) administration of peptides.

### Behavioral Analysis

Behavioral experiments were performed between 09:00 and 14:00 h and rats, once used, were not reemployed. Animals were adapted to handling and were taken into the experimental room (80–90 lux) for at least 1 h to reach habituation before the icv peptide administration. Doses for GAL, the NPYY1R agonist [Leu^31^,Pro^34^]NPY and for GALR2 antagonist M871 were chosen based on previous dose-response curves (Bing et al., 1993; Broqua et al., 1995; Narvaez et al., 2015, 2016). Rats were individually placed and allowed to freely explore the behavioral apparatus over a 5 min period. Activity was analyzed using the Raton Time 1.0 software (Fixma S.L., Valencia, Spain). Locomotor parameters were analyzed using the video tracking software Smart2.5 (Panlab). After each trial, all surfaces were cleaned with 70% ethanol solution. Behaviors scored in the open field, elevated plus maze and light-dark box were: Rearing time and frequency (either with the animal propped up on its hind limbs with the forepaws in contact with the sides of the task or nothing at all), stretch attends postures (SAP) time and frequency (the rodent lowers its back, elongates its body and is either standing still or moving forward very slowly) and freezing time and frequency (cessation of movement). Head-dipping behavior in the elevated plus maze (with the rat looking down over the edges of its open arms) and the latency to enter (with all four paws) to the dark compartment in the light-dark box were also studied. Open field, elevated plus maze and light–dark box were performed as previously described (Millon et al., 2015; Narvaez et al., 2015). All behavioral experiments were carried out by observers blinded to all experimental conditions.

### c-Fos Immunohistochemistry

Anesthetized rats with sodium pentobarbital (Mebumal; 100 mg/kg, i.p.) were perfused with 4% paraformaldehyde (wt/vol, Sigma) 90 min after icv injections and brains were coronally sliced and immunostained.

Animals were divided into five experimental groups: (1) aCSF: control group; (2) GAL-treated group (3 nmol); (3) Y1-treated group receiving an NPYY1R agonist [Leu^31^,Pro^34^]NPY (2.5 nmol); (4) GAL+Y1: group administered with both substances; and (5) GAL+Y1+M871: group injected with GAL, [Leu^31^,Pro^34^]NPY and the GALR2 antagonist (M871; 3 nmol) (*N* = 4 in each group). Doses indicated above and the c-Fos procedure is based on previously published protocols (Narvaez et al., 2015, 2016).

As primary antibodies, an antibody against the c-Fos protein (1:5000, sc-52, Santa Cruz Biotechnology, CA), revealed with DAB plus nickel, was used as an indirect marker of neural activity. A second primary antibody was used for Calbindin-D28k (1:1000, Santa Cruz Biotechnology, CA), revealed with DAB. Double immunohistochemistry with Calbindin allow to outline subregions [i.e., medial paracapsular intercalated (ITCp) clusters in the amygdala are easy to be identified from the rest of the darkly stained amygdaloid areas] or to characterize neuronal populations (i.e., orexin neurons in perifornical hypothalamic región) (Kemppainen and Pitkanen, 2000; Scharfman et al., 2002). Complementary, we detected the position of ITCp cell clusters in the amygdala on adjacent sections stained with 0,1% cresyl violet, using accepted cytoarchitectonic criteria (Swanson, 1992). Appropriate biotinylated specific secondary antibodies were used. Sections were mounted on glass slides and the different amygdala, hypothalamic and PAG subregions were analyzed using the optical fractionator method in unbiased stereological microscopy (Olympus BX51 Microscope, Olympus, Denmark) as previously described (see Supplement Material for details).

### *In Situ* Proximity Ligation Assay

*In situ* PLA was performed as previously described (Borroto-Escuela et al., 2013; Narvaez et al., 2016). Treated rats were divided into experimental groups: (1) aCSF: control group; (2) GAL-treated group (3 nmol); (3) Y1-treated group receiving an NPYY1R agonist [Leu^31^,Pro^34^]NPY (3 nmol); (4) GAL+Y1: group administered with both substances; and (5) GAL+Y1+M871: group injected with GAL, [Leu^31^,Pro^34^]NPY and the GALR2 antagonist (M871; 3 nmol). (*N* = 4 in each group). Animals were perfused with 4% paraformaldehyde 24 h after icv injections, brains were removed and sections were obtained.

Knockdown GALR2 siRNA rats were generated and verified their effectiveness (Millon et al., 2015; Flores-Burgess et al., 2017). Using quantification of immunohistochemical staining and real-time quantitative PCR we performed a time course of GALR2 mRNA and GALR2 protein expression. The time course curve indicated a maximal reduction of GALR2 receptor protein expression 8 days after the injection. Briefly, during the stereotaxic surgery, once the cannula is fixed, animals received an intracerebroventricular (icv) injection of 5 mg (0.35 nmol) of Accell Smart pool siRNA for GALR2 (Dharmacon). Animals had a recovery period after 8 days, the time required to reduce the levels of GALR2. For PLA analysis Knockdown GALR2 group (Accell siRNA GALR2) was compared with the Vehicle group (Accell siRNA Delivery Media) (*N* = 4 in each group), since no differences were observed with siRNA Control rats (Accell non-targeting pool) (Millon et al., 2015; Flores-Burgess et al., 2017).

Free-floating sections were incubated with blocking (5% goat serum) and permeabilization (0.3% Triton X100 in PBS) solutions during 60 min each. Primary antibodies of different hosts directed against GALR2 (rabbit, Alomone Lab, 1:100) and NPYY1R (goat, sc-21992 Santa Cruz Biotechnology, Inc., CA, 1:200) were incubated for 24 h at 4°C. PLA signal detection was performed according to manufacturer’s instructions (Duolink *in situ* PLA detection kit; Olink, Sweden) with PLA PLUS or MINUS probes for rabbit or goat antibodies. Sections were mounted on slides with mounting medium (Dako) containing 4′,6-diamidino-2- phenylindole (DAPI) (1:200), staining nuclei with blue color. Control experiments used only one primary antibody. PLA signals were visualized by using a TCS-SL confocal microscope (Leica).

### Statistical Analysis

Data are expressed as mean ± SEM, and sample number (*n*) is indicated in figure legends. All data were analyzed using GraphPad PRISM 6.0 (GraphPad Software, La Jolla, CA, United States).

For comparing two experimental conditions, Student’s unpaired *t*-test statistical analysis was performed. Otherwise, one-way analysis of variance (ANOVA) followed by the Newman–Keuls comparison *post hoc* test was performed. Differences were considered significant at *p* < 0,05 (^∗^*p* < 0.05, ^∗∗^*p* < 0.01, ^∗∗∗^*p* < 0.001).

## Results

### Behavioral Profiles Induced in the Open Field, Elevated Plus-Maze and Light-Dark Box by GALR2/NPYY1R Interactions.

#### Open Field

**FIGURE 1:**
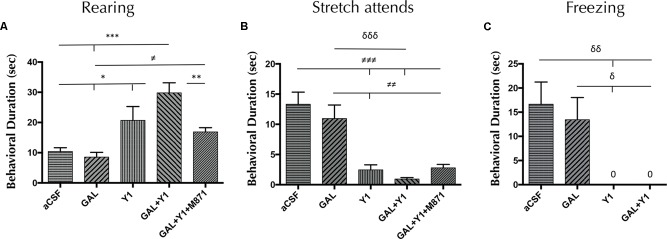
Open field behavioral analysis mediated by Galanin (GAL) and the Neuropeptide Y Y1 receptor (NPYY1R) agonist. The response induced by GAL on the NPYY1-mediated ethological behaviors is blocked by the GAL receptor 2 (GALR2) antagonist M871. Cumulative behavioral duration of rearings **(A)**, stretch attends postures (SAP) **(B)** and freezing **(C)** in the open field. Data represent mean ± SEM. *N* = 6–8 animals in each group. Value for GAL+Y1+M871 group on freezing was 0.07 ± 0.06. ^∗^*P* < 0.05; ^∗∗^*P* < 0.01; ^∗∗∗^*P* < 0.001; ^≠^*P* < 0.05; ^≠≠^*P* < 0.01; ^≠≠≠^*P* < 0.001; ^δ^*P* < 0.05; ^δδ^*P* < 0.01; ^δδδ^*P* < 0.001 according to one-way ANOVA followed by Newman–Keuls Multiple Comparison Test. Inter-group comparisons are indicated by the lines above bars. GAL, Galanin 3 nmol; Y1 = NPY receptors agonist [Leu^31^-Pro^34^]NPY 3 nmol; GAL+Y1, Coadministration of GAL and Y1; GAL+Y1+M871, Coadministration of GAL, Y1 and GALR2 antagonist M871 3 nmol.

In the open field, the intracerebroventricular (icv) administration of the NPYY1R agonist at 3 nmol increased the time of rearings (one-way ANOVA, F4, 25 = 12.08, *p* < 0.001, Newman–Keuls *post hoc* test: *p* < 0.05), decreased time of the SAP (one-way ANOVA, F4, 29 = 13.02, *p* < 0.001, Newman–Keuls *post hoc* test: *p* < 0.001) and time of freezing (one-way ANOVA, F4, 29 = 6.03, *p* < 0.01, Newman–Keuls *post hoc* test: *p* < 0.01) compared with control animals (**Figure 1**). Regarding frequency, the NPYY1R agonist injection increased rearings (one-way ANOVA, F4, 24 = 10.5, *p* < 0.001, Newman–Keuls *post hoc* test: *p* < 0.05) and decreased both, SAP (one-way ANOVA, F4, 29 = 10.6, *p* < 0.001, Newman–Keuls *post hoc* test: *p* < 0.001) and freezing (one-way ANOVA, F4, 30 = 11.3, *p* < 0.001, Newman–Keuls *post hoc* test: *p* < 0.05) episodes compared with control group (Supplementary Figure 1). GAL at 3 nmol lacked effects on all the parameters analyzed (**Figure 1** and Supplementary Figure 1). However, a specific enhancement of time of rearing behavior (Newman–Keuls *post hoc* test: *p* < 0.05) was observed after the coadministration of GAL and the NPYY1R agonist compared with the NPYY1R agonist alone (**Figure 1**). Moreover, GAL and NPYY1R agonist coinjection also enhanced the frequency on rearing (Newman–Keuls *post hoc* test: *p* < 0.05) (Supplementary Figure 1). There was also observed following the coadministration of both peptides a non-significant trend to decrease SAP compared with NPYY1R agonist alone (**Figure 1** and Supplementary Figure 1). The involvement of GALR2 in this interaction was validated since the presence of the GALR2 antagonist M871 counteracted the enhancement of both time and frequency on rearing behavior induced by the coadministration of GAL and NPYY1R agonist (**Figure 1** and Supplementary Figure 1). No differences were observed between groups in locomotor parameters (Supplementary Table 3).

#### Elevated Plus-Maze

**FIGURE 2:**
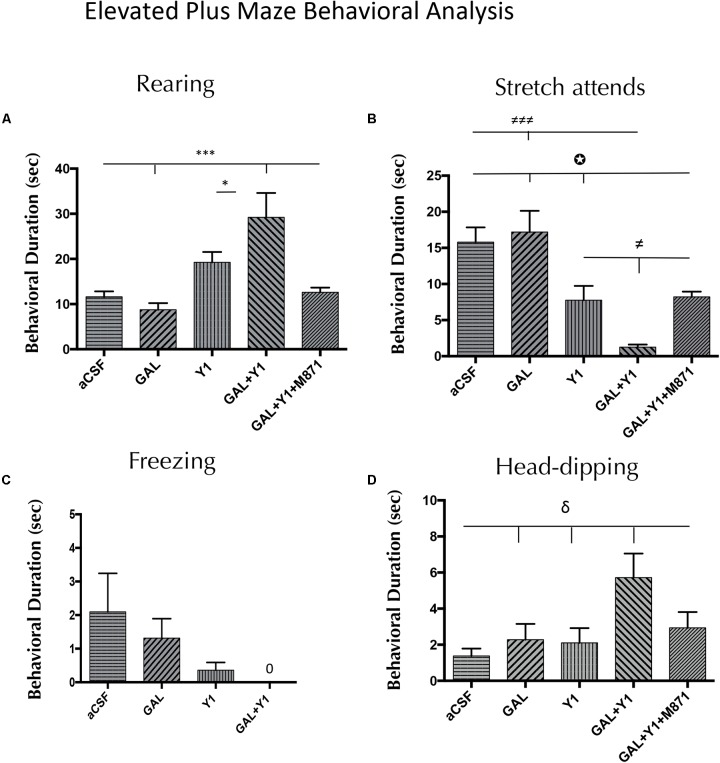
Behavioral analysis mediated by Galanin (GAL) and the NPYY1R agonist in the elevated plus maze. The response induced by GAL on the NPYY1-mediated ethological behaviors is blocked by the GAL 2 receptor (GALR2) antagonist M871. Cumulative behavioral duration of rearings **(A)**, SAP **(B)**, freezing **(C)** and head-dipping **(D)** in this test. *N* = 6–8 animals in each group. Data represents mean ± SEM. Value for GAL+Y1+M871 group on freezing was 0. ^∗^*P* < 0.05; ^∗∗∗^*P* < 0,001; ^≠^*P* < 0.05; ^≠≠≠^*P* < 0.001; ^δ^*P* < 0.05; 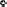
*P* < 0.05 according to one-way ANOVA followed by Newman–Keuls Multiple Comparison Test. Inter-group comparisons are indicated by the lines above bars. GAL, Galanin 3 nmol; Y1 = NPY Y1 receptors agonist [Leu^31^-Pro^34^]NPY 3 nmol; GAL+Y1, Coadministration of GAL and Y1; GAL+Y1+M871, Coadministration of GAL, Y1 and GALR2 antagonist M871 3 nmol.

In the elevated plus-maze, a significant increase in time spent on rearing behavior (one-way ANOVA, F4, 25 = 8.58, *p* < 0.001, Newman–Keuls *post hoc* test: *p* < 0.05) and having head-dipping (one-way ANOVA, F4, 28 = 3.91, *p* < 0.05, Newman–Keuls *post hoc* test: *p* < 0.05) was observed after the coadministration of GAL and the NPYY1R agonist, compared with NPYY1R agonist alone (**Figure 2**). Furthermore, after GAL and NPYY1R agonist coinjection was observed a significant increase of frequency in rearing behavior (one-way ANOVA, F4, 27 = 4.47, *p* < 0.05, Newman–Keuls *post hoc* test: *p* < 0.05) and head-dipping (one-way ANOVA, F4, 28 = 5.21, *p* < 0.01, Newman–Keuls *post hoc* test: *p* < 0.01) compared with NPYY1R agonist alone (Supplementary Figure 2). Moreover, decreased time (one-way ANOVA, F4, 27 = 11.64, *p* < 0.001, Newman–Keuls *post hoc* test: *p* < 0.05) and frequency (one-way ANOVA, F4, 27 = 13.64, *p* < 0.001, Newman–Keuls *post hoc* test: *p* < 0.001) of SAP were also noticed by GAL and NPYY1R agonist coinjection (**Figure 2** and Supplementary Figure 2). GALR2 participated in these interactions since the presence of the GALR2 antagonist M871 blocked the effects induced by the coadministration of GAL and NPYY1R agonist (**Figure 2** and Supplementary Figure 2). However, the behavioral profile of the NPYY1R agonist alone was slightly different compared with open field, with no significant effects on rearing and inducing only a decreased time (Newman–Keuls *post hoc* test: *p* < 0.05) on SAP in the elevated plus-maze. Similarly as described above, GAL injections alone lacked effect in all the behaviors analyzed (**Figure 2** and Supplementary Figure 2). No differences were observed between groups in locomotor parameters (Supplementary Table 3).

#### Light-Dark Box

**FIGURE 3:**
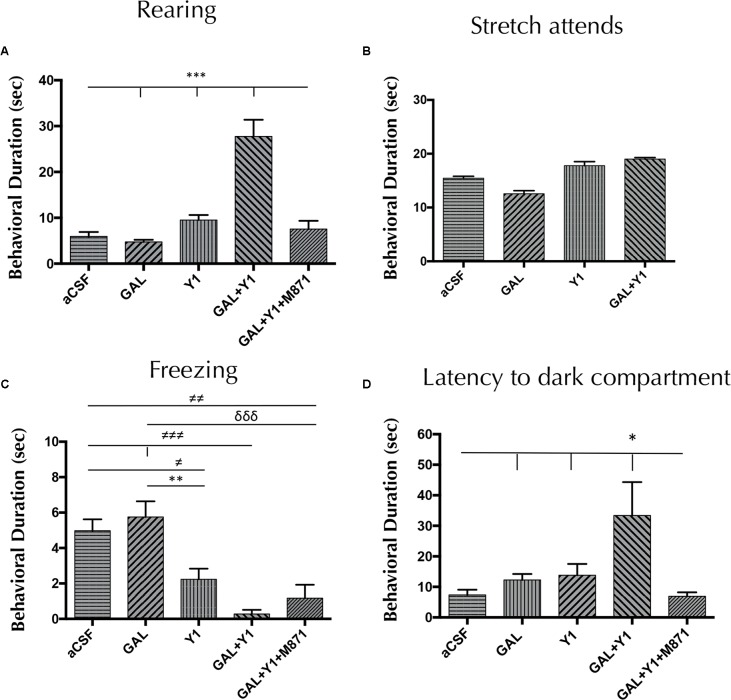
Light-dark box behavioral analysis mediated by Galanin (GAL) and the NPYY1R agonist. The response induced by GAL on the NPYY1-mediated ethological behaviors is blocked by the GAL 2 receptor (GALR2) antagonist M871. Cumulative behavioral duration on rearings **(A)**, SAP **(B),** freezing **(C)** and latency to enter to the dark compartment **(D)** in the light-dark box. *N* = 6–8 animals in each test group. Data represent mean ± SEM. Value for GAL+Y1+M871 group on stretch attends was 16.3 ± 0.59. ^∗^*P* < 0.05; ^∗∗^*P* < 0.01; ^∗∗∗^*P* < 0.001; ^≠^*P* < 0.05; ^≠≠^*P* < 0.01; ^≠≠≠^*P* < 0.001; ^δδδ^*P* < 0.0001 according to one-way ANOVA followed by Newman–Keuls Multiple Comparison Test. Inter-group comparisons are indicated by the lines above bars. GAL, Galanin 3 nmol; Y1, NPY Y1 receptors agonist [Leu^31^-Pro^34^]NPY 3 nmol; GAL+Y1, Coadministration of GAL and Y1; GAL+Y1+M871, Coadministration of GAL, Y1 and GALR2 antagonist M871 3 nmol.

As in the elevated plus-maze, in the light-dark box a specific increase of the time (one-way ANOVA, F4, 26 = 21.82, *p* < 0.001, Newman–Keuls *post hoc* test: *p* < 0.001) of rearing behavior was observed after the GAL and NPYY1R agonist coinjection, compared with both peptides alone (**Figure 3**). In a similar way, GAL and NPYY1R agonist coinjection increased frequency (one-way ANOVA, F4, 26 = 4.2, *p* < 0.01, Newman–Keuls *post hoc* test: *p* < 0.05) on rearing behavior, compared with both peptides alone (Supplementary Figure 3). Again, GALR2 seems crucial for this interaction, since coadministration of the GALR2 antagonist M871 counteracted the enhancement of both parameters on rearing behavior (**Figure 3** and Supplementary Figure 3). In addition, a tendency for suppressing time and frequency on freezing behavior was observed after GAL and NPYY1R agonist coinjection compared with NPYY1R agonist alone (**Figure 3** and Supplementary Figure 3). Latency (one-way ANOVA, F4, 22 = 4.39, *p* < 0.01, Newman–Keuls *post hoc* test: *p* < 0.05) to enter to the dark compartment was increased by GAL and NPYY1R agonist coinjection, compared with the two peptides given alone (**Figure 3**). NPYY1R agonist alone only decreased time (one-way ANOVA, F4, 23 = 7.61, *p* < 0.001, Newman–Keuls *post hoc* test: *p* < 0.01) and frequency (one-way ANOVA, F4, 27 = 16.1, *p* < 0.001, Newman–Keuls *post hoc* test: *p* < 0.05) of freezing behavior, compared with aCSF (**Figure 3** and Supplementary Figure 3). No differences were observed between groups in locomotor parameters (Supplementary Table 3).

### GAL and NPYY1R Agonist Coadministration Showed a Specific c-Fos Activation Pattern in the Amygdala-Hypothalamus-PAG Axis

#### Medial Paracapsular Intercalated Nucleus

**FIGURE 4:**
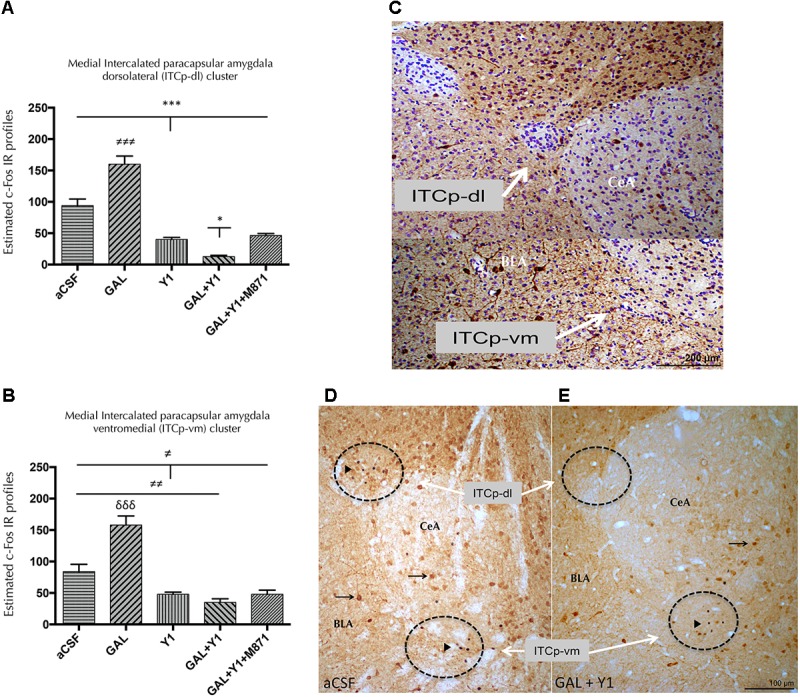
Effects of Galanin (GAL) and NPYY1R agonist, alone in combination or together with the GAL 2 receptor (GALR2) antagonist M871 on c-Fos expression in the medial intercalated paracapsular amygdaloid (ITCp) islands. Quantification of total c-Fos IR nuclei on ITCp, in dorsolateral (ITCp-dl) **(A)** and ventromedial (ITCp-vm) **(B)** clusters. Data, expressed as mean ± SEM show the differences between groups after the icv injection of aCSF, GAL, NPYY1 agonist [Leu^31^-Pro^34^]NPY, or the coadministration of both peptides and M871. **(A)** The coadministration of GAL and NPYY1 agonist decreased c-Fos expression specifically in the ITCp-dl as compared with the effects both peptides alone and the aCSF group. Moreover, the effects of GAL and NPYY1 agonist coadministration were counteracted by the GALR2 antagonist M871. **(B)** The administration of the NPYY1 agonist decreased the c-Fos expression in the ITCp-vm in a similar way compared including its combination with GAL. ^∗^*P* < 0.05; ^∗∗∗^*P* < 0.001; ^≠^*P* < 0.05; ^≠≠^*P* < 0.01; ^≠≠≠^ *P* < 0.001; ^δδδ^ *P* < 0.001 according to one-way ANOVA followed by Newman–Keuls Multiple Comparison Test. Inter-group comparisons are indicated by the lines above bars. *N* = 4 in each group. **(C)** Digital photo-montage illustrating the analyzed ITCp dorsolateral (ITCp-dl) and ventromedial (ITCp-vm) clusters located between basolateral (BLA) and central (CeA) amygdaloid nucleus [Bregma: –2.3 mm; according to the Paxinos and Watson (1986) stereotaxical atlas]. Sections were double immunostained with Calbindin and counterstained with cresyl violet. The coadministration of GAL and NPYY1R agonist is illustrated to decrease the c-Fos expression especially in non-calbindinergic neurons specifically in the ITCp-dl compared with the control group **(D,E)**. White arrows indicate ITC-dl and ITCp-vm cell clusters. Black arrowheads indicate neurons that are c-Fos+/Calbindin-; black arrows indicate neurons that are c-Fos-/Calbindin+. Dashed circles represent measuring fields. Abbreviations: aCSF, cerebrospinal fluid; GAL = Galanin 3 nmol; Y1, NPY Y1 receptor agonist [Leu^31^-Pro^34^]NPY 2,5 nmol; GAL+Y1, Coadministration of GAL and [Leu^31^-Pro^34^]NPY; GAL+Y1+M871, Coadministration of GAL, [Leu^31^-Pro^34^]NPY and GALR2 antagonist M871 3 nmol.

To determine the subregions of the medial paracapsular intercalated (ITCp) subnuclei of the amygdala involved in the above effects, double immunohistochemistry was performed to determine the expression of the immediate early gene Fos (c-Fos IR) and calbindin. The icv injection of [Leu^31^,Pro^34^]NPY alone induced a decrease in the number of c-Fos IR profiles in the dorsolateral (ITCP-dl) (one-way ANOVA, F4, 17 = 64.7, *p* < 0.001, Newman–Keuls *post hoc* test: *p* < 0.001) (**Figures 4A,C**) and ventromedial (ITCp-vm) (one-way ANOVA, F4, 16 = 31.29, *p* < 0.001, Newman–Keuls *post hoc* test: *p* < 0.05) (**Figures 4B,C**) subregions of the ITCp, compared with aCSF group, respectively. On the contrary, GAL alone significantly increased the number of c-Fos IR profiles in both areas, the ITCp-dl (Newman–Keuls *post hoc* test: *p* < 0.001) and ITCp-vm (Newman–Keuls *post hoc* test: *p* < 0.001) compared with the aCSF groups (**Figures 4A–C**). However, GAL and NPYYR1 agonist coinjection significantly decreased the number of c-Fos IR profiles specifically in the ITCp-dl compared with NPYY1R agonist alone (Newman–Keuls *post hoc* test: *p* < 0.05) (**Figures 4A–E**). The cotreatment with the GALR2 antagonist M871 completely reversed the GAL contribution to the response in the ITCp-dl subregion (**Figure 4A**), demonstrating the involvement of GALR2 in the GAL/NPYY1R agonist actions.

#### Ventromedial Hypothalamic Nucleus

**FIGURE 5:**
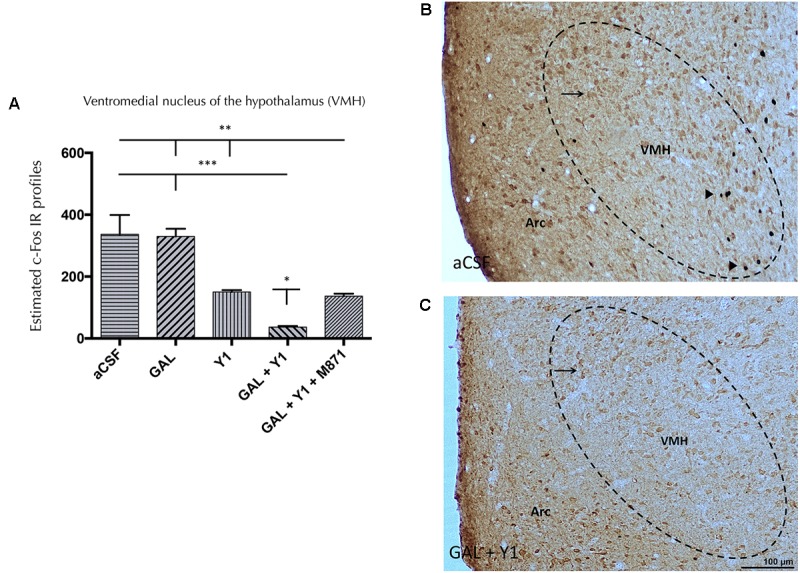
c-Fos expression in ventromedial hypothalamic (VMH) nucleus after the intracerebroventricular (icv) administration of Galanin (GAL) and NPYY1R agonist, either alone or in combination together with the GAL 2 receptor (GALR2) antagonist M871. Quantification of total c-Fos IR nuclei in VMH **(A)**. Data, expressed as mean ± SEM showed the differences between groups after icv injection of aCSF, GAL, [Leu^31^-Pro^34^]NPY, or the coadministration of both peptides and M871. *N* = 4 in each group. **(A)** GAL and the NPYY1 agonist coinjection decreased the c-Fos expression in the VMH compared with the effects of both peptides alone and the aCSF group. Furthermore, this effect was blocked by GALR2 antagonist M871. ^∗^*P* < 0.05; ^∗∗^*P* < 0.01; ^∗∗∗^*P* < 0.001 according to one-way ANOVA followed by Newman–Keuls Multiple Comparison Test. Inter-group comparisons are indicated by the lines above bars. *N* = 4 in each group. Sections were double immunostained with Calbindin. GAL and NPYY1R agonist coinjection **(C)** decreased the c-Fos expression in non-calbindinergic neurons in the VMH compared with the control group **(B)**. Black arrowheads indicate neurons that are c-Fos+/Calbindin-; black arrows indicate neurons that are c-Fos-/Calbindin+. Dashed ovals represent measuring fields in VMH near the arcuate (Arc) hypothalamic nucleus (Bregma: –2.56 mm). Abbreviations: aCSF, cerebrospinal fluid; GAL, Galanin 3 nmol; Y1, NPY Y1 receptor agonist [Leu^31^-Pro^34^]NPY 2,5 nmol; GAL + Y1, Coadministration of GAL and [Leu^31^-Pro^34^]NPY; GAL+Y1+M871, Coadministration of GAL, [Leu^31^-Pro^34^]NPY and GALR2 antagonist M871 3 nmol.

In the ventromedial nucleus of the hypothalamus (VMH), a similar c-Fos IR pattern was observed after the coadministration of NPYYR1 agonist and GAL (**Figure 5**). The coinjection of GAL and NPYYR1 agonist significantly decreased (one-way ANOVA, F4, 15 = 18.35, *p* < 0.001, Newman–Keuls *post hoc* test: *p* < 0.05) the number of c-Fos IR profiles, compared to the effect of NPYY1R agonist alone, in the VMH. Again, the presence of the GALR2 antagonist M871 completely reversed this decrease (**Figure 5**), demonstrating the involvement of GALR2 in this interaction. While the injection of GAL alone lacked effects, the icv injection of NPYY1R agonist alone induced a decrease in the number of c-Fos IR profiles in the VMH (Newman–Keuls *post hoc* test: *p* < 0.01) (**Figure 5**).

#### Perifornical Hypothalamic Nucleus

**FIGURE 6:**
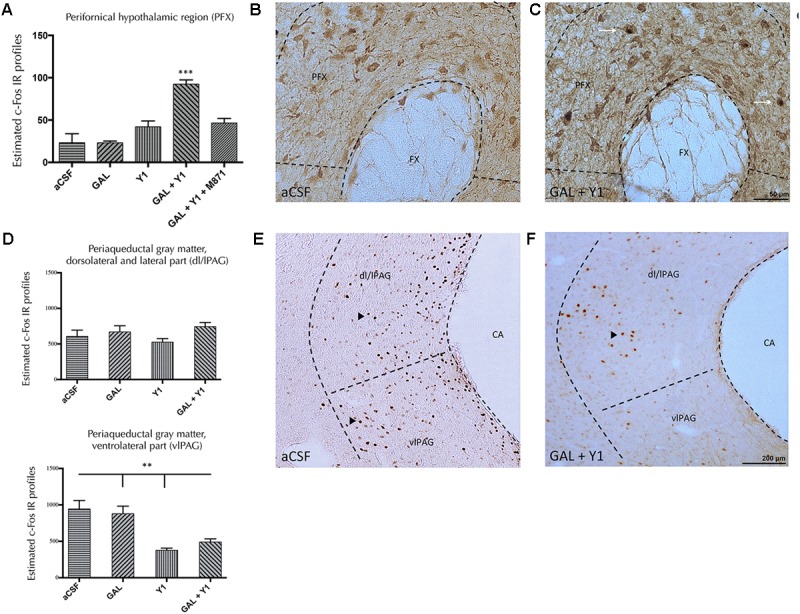
Effects of Galanin (GAL) and NPYY1R agonist alone, together or in combination with the GAL 2 receptor (GALR2) antagonist M871, on c-Fos expression in the perifornical region (PFX) and periaqueductal gray (PAG) region. Quantification of total c-Fos IR nuclei in PFX **(A)** and PAG, dorsolateral/lateral (dl/lPAG) **(D**, Top**)** and ventromedial (vmPAG) **(D**, Bottom**)** subregions. Data, expressed as mean ± SEM show the differences between groups after intracerebroventricular injections of aCSF, GAL, [Leu^31^-Pro^34^]NPY, both peptides or the coadministration of both peptides and M871. **(A)** The coadministration of GAL and the NPYY1 agonist increased the c-Fos expression in the PFX compared with the infusion of each peptide alone and the aCSF group. Moreover, this effect was blocked by the GALR2 antagonist M871. ^∗∗∗^*P* < 0.001 GAL+Y1 versus the rest of the groups according to one-way ANOVA followed by Newman–Keuls Multiple Comparison Test. *N* = 4 in each group. Sections were double immunostained with Calbindin. GAL and NPYY1R agonist coinjection **(C)** increased the c-Fos expression in calbindinergic neurons in the PFX compared with the control group **(B)**. Dashed lines represent measuring fields around upper region of fornix (FX). White arrows indicate neurons that are c-Fos+/Calbindin+. [Bregma: –3.14 mm; according to the Paxinos and Watson (1986) stereotaxical atlas]. After infusion of GAL, NPYY1 agonist or the combination no differences were observed on c-Fos IR profiles in the dl/lPAG **(D**, Top**)**. However, NPYY1 agonist and the coadministration with GAL decreased c-Fos expression specifically in the vlPAG compared with GAL alone and the aCSF group **(D**, Bottom**)**. ^∗∗^*P* < 0.01 according to one-way ANOVA followed by Newman–Keuls Multiple Comparison Test. Inter-group comparisons are indicated by the lines above bars. C-Fos IR value for GAL+Y1+M871 group on dl/lPAG was 543.8 ± 63.6 and on vmPAG 498 ± 44.57. Representative microphotographs show how the coadministration of both peptides GAL and NPYY1R agonist **(F)** decreased the c-Fos expression in the vlPAG compared with the control group **(E)**. Dashed lines represent measuring fields around the cerebral aqueduct (CA). Black arrowheads indicate c-Fos IR profiles. [Bregma: –7.8 mm; according to the Paxinos and Watson (1986) stereotaxical atlas]. Abbreviations: aCSF, cerebrospinal fluid; GAL, Galanin 3 nmol; Y1, NPY Y1 receptor agonist [Leu^31^-Pro^34^]NPY 2,5 nmol; GAL+Y1, Coadministration of GAL and [Leu^31^-Pro^34^]NPY; GAL+Y1+M871, Coadministration of GAL, [Leu^31^-Pro^34^]NPY and GALR2 antagonist M871 3 nmol.

In the perifornical nucleus of the hypothalamus (PFX), the injection of GAL or the NPYY1R agonist alone lacked effects on the c-Fos IR profiles (**Figure 6A**). However, the coinjection of GAL and NPYYR1 agonist significantly increased (one-way ANOVA, F4, 15 = 18.45, *p* < 0.001, Newman–Keuls *post hoc* test: *p* < 0.001) the number of c-Fos IR profiles, compared with GAL or the NPYY1R agonist alone. Increased c-Fos IR was observed mainly in calbininergic neurons within PFX region (**Figures 6A–C**). Also, the coadministration with the GALR2 antagonist M871 completely restored this increase (**Figure 6A**), demonstrating again the involvement of GALR2 in this interaction.

#### Periaqueductal Gray

Within the PAG region we analyzed the dorsolateral and lateral (dl/lPAG) and the ventrolateral parts (vlPAG) (**Figure 6D**). No modifications on c-Fos IR were observed after the injections of GAL, NPYY1R agonist alone or following their coinjection in the dl/lPAG region. However, the injection of the NPYY1R agonist alone (one-way ANOVA, F4, 15 = 11.26, *p* < 0.001, Newman–Keuls *post hoc* test: *p* < 0.01) or coinjected with GAL (Newman–Keuls *post hoc* test: *p* < 0.01) significantly decreased c-Fos IR profiles in the vlPAG region (**Figures 6D–F**), compared with aCSF and GAL.

Lack of c-Fos IR modifications was observed in the paraventricular nucleus of the hypothalamus (PVN) after GAL, NPYY1R agonist or their combination (Supplementary Table 1). There was also observed after GAL and NPYY1R coadministration a tendency to decrease c-Fos IR in the medial part of the central (CeM) amygdala, but without statistical significance (Supplementary Table 1).

Moreover, no changes were detected on plasma corticosterone levels after open field or elevated plus-maze induced either by the sole administration of GAL, NPYY1R agonist or following their coinjection (Supplementary Table 2).

### GALR2/NPYY1R Heteroreceptor Complexes Increase Within ITCp-dl Upon Agonist Coactivation of GALR2 and NPYY1R

To analyze the region-specific GALR2/NPYY1R heteroreceptor complexes formation within the ITCp islands we performed *in situ* proximity ligation assay (PLA), observing the dorsolateral (ITCp-dl) and ventromedial part (ITCP-vm) of the ITCp.

**FIGURE 7:**
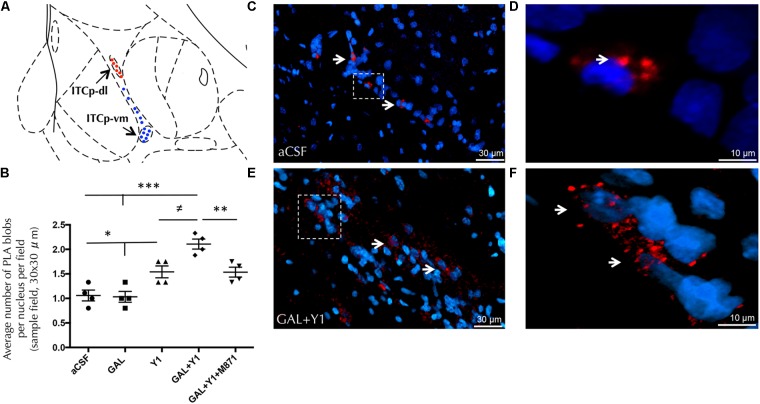
Detection of GALR2/NPYY1R heteroreceptor complexes with *in situ* PLA in the dorsolateral cluster of medial paracapsular intercalated islands of the amygdala (ITCp-dl). **(A)** Diagram shows the presence of positive PLA signals (red circles) in the ITCp-dl and lack of specific signals (blue circles) in the ventromedial cluster (ITCp-vm). [Bregma: –2.3 mm; according to the Paxinos and Watson (1986) stereotaxical atlas]. **(B)** Quantification of PLA signals in ITCp-dl was performed by measuring blobs per nucleus per sampled field by an experimenter blind to treatment conditions. Sprague Dawley rats showed a significant increase in GALR2/NPYY1R heteroreceptor complexes (PLA blobs) in the ITCp-dl cluster after GAL and NPYY1 agonist coinjection. This effect was blocked by the presence of the GALR2 antagonist M871. ^∗^*P* < 0.05; ^∗∗^*P* < 0.01; ^∗∗∗^*P* < 0.001; ^≠^*P* < 0.05 according to one-way ANOVA followed by Newman–Keuls Multiple Comparison Test. Inter-group comparisons are indicated by the lines above bars. Data are expressed as mean ± SEM and each point in the graph represents results from individual rats from multiple photographs analyzed (*N* = 4 rats per group). **(C–F)** Representative microphotographs of the significant increase in the density of GALR2/NPYY1R positive red PLA blobs in the ITCp-dl subregion after GAL and NPYY1 agonist coinjection **(E)** compared with control group **(C)**. Magnified views from dashed boxes in **C,E** are shown in **(D)** and **(F),** respectively. GALR2/NPYY1R heteroreceptor complexes are shown as red PLA blobs (clusters) found in high densities per cell in a large number of nerve cells using confocal laser microscopy, White arrows point to PLA clusters. The nuclei are shown in blue by DAPI. Abbreviations: aCSF, cerebrospinal fluid; GAL, Galanin 3 nmol; Y1, NPY Y1 receptor agonist [Leu^31^-Pro^34^]NPY 3 nmol; GAL+Y1, Coadministration of GAL and [Leu^31^-Pro^34^]NPY; GAL+Y1+M871, Coadministration of GAL, [Leu^31^-Pro^34^]NPY and GALR2 antagonist M871 3 nmol.

PLA-positive red clusters were found specifically in cells of the ITCp-dl subregion, compared with some scattered PLA signals in the ITCp-vm (**Figure 7A** and Supplementary Figure 4). Quantification of PLA demonstrated an increase in the density of the PLA-positive red clusters (one-way ANOVA, F4, 15 = 16.23, *p* < 0.001, Newman–Keuls *post hoc* test: *p* < 0.05) after NPYY1R agonist injection compared to control or GAL injections (**Figure 7B**). Moreover, the coinjection of GAL and NPYYR1 agonist significantly increased (Newman–Keuls *post hoc* test: *p* < 0.05) the number of PLA-positive red clusters within the ITCp-dl (**Figures 7B–F**) compared with NPYYR1 agonist alone. Similarly to the c-Fos response described above, the presence of the GALR2 antagonist M871 completely blocked this increase (**Figure 7B**), demonstrating the involvement of GALR2 in this interaction.

**FIGURE 8:**
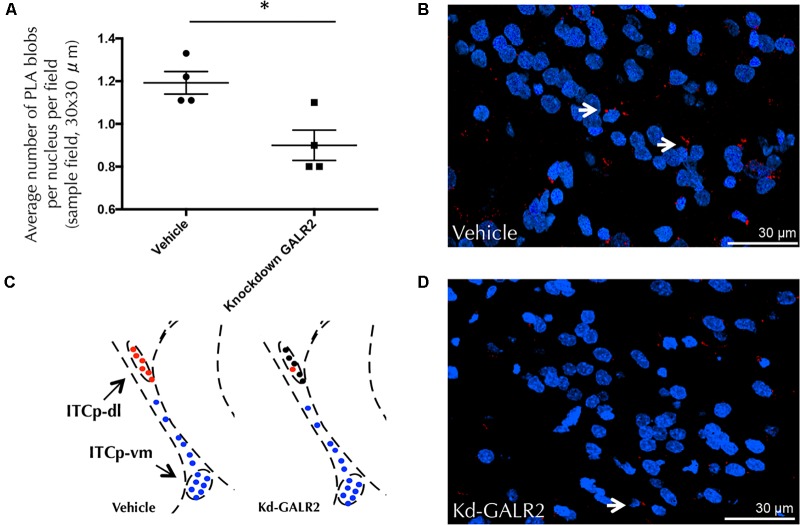
GALR2/NPYY1R heteroreceptor complexes are modified in the dorsolateral cluster of the medial paracapsular intercalated nucleus of the amygdala (ITCp-dl) by using *in situ* PLA after knockdown of GALR2 in rats. No differences were observed with siRNA Control rats (Accell Non-targeting pool) (Millon et al., 2015; Flores-Burgess et al., 2017). Therefore, vehicle group (Accell siRNA Delivery Media) was used as control. **(A)** Quantification of PLA signals as blobs per nucleus per sampled field by an experimenter blind to treatment conditions. Knockdown GALR2 rats showed a significant decrease in GALR2/NPYY1R heteroreceptor complexes (PLA blobs) in the ITCp-dl cluster. ^∗^*P* < 0.05 knockdown (Kd) GALR2 versus vehicle rats according to Student’s *t*-test. Data are expressed as mean ± SEM, Each point in the graph represents results from multiple photographs analyzed and taken from individual rats (*N* = 4 rats per group). **(B)** Diagram shows positive PLA signals (red circles) in the ITCp-dl in control animals and loss of specific signals (black circles) in the ITCp-dl in knockdown GALR2 rats. Blue circles indicate lack of specific signals in the ventromedial cluster (ITCp-vm). [Bregma: –2.3 mm; according to the Paxinos and Watson (1986) stereotaxical atlas]. **(C,D)** Representative microphotograph of the decreased density of GALR2/NPYY1R positive red PLA blobs observed in the GALR2 Knockdown animals **(D)** compared with control rats **(C)**. GALR2/NPYY1R heteroreceptor complexes are shown as red PLA blobs (clusters) using confocal laser microscopy, White arrows point to red PLA clusters. The nuclei are shown in blue by DAPI. Abbreviations: Control, Rats injected icv with Accell siRNA Delivery Media; Kd-GALR2, Rats injected icv with Accell siRNA GALR2.

Furthermore, siRNA GALR2 knockdown rats were used to validate the GALR2 involvement on GALR2/NPYY1R heteroreceptor complexes in the ITCp-dl subregion. Quantification of PLA-positive red clusters in the GALR2 knockdown rats demonstrated a reduction of PLA-positive signals (*t* = 3.32, *p* < 0,05, df = 6) compared with the control group (**Figure 8**). No specific PLA-positive red clusters were observed neither in the hypothalamic nor PAG regions studied in c-Fos experiments.

## Discussion

The current study confirms the existence of an interaction between GALR2 and NPYY1R seen upon combined receptor agonist treatment acting at these receptors. This GALR2 and NPYY1R coactivation elicited in innate models of anxiety a specific behavioral profile on ethological parameters, increasing rearing and head-dipping and reducing stretch attend postures and freezing, that strongly support anxiolytic actions. This anxiolytic effect seems to be linked to the ability of the coagonist treatment to significantly increase the density of GALR2/NPYY1R heteroreceptor complexes in the ITCp-dl subregion of the amygdala. This event may contribute to the ability of GALR2 and NPYY1R coactivation to enhance the NPYY1R-mediated reduction in the number of c-Fos IR profiles in this intercalated region (ITCp-dl) and thus their neuronal and circuit activity modifications. Previously, we demonstrated c-Fos modifications on GABA interneurons, marked with GAD 65/67, in intercalated amygdala induced by GALR2 and NPYY1R coactivation (Narvaez et al., 2015). So, it is proposed that the mechanism that underlies the anxiolytic actions was mediated by disinhibition of GABA ITCp-vm interneurons in the circuit controlling the GABA inhibition of the anxiogenic outflow from medial subnucleus of the central amygdala (Perez de la Mora et al., 2008). The significant c-Fos decrease in the VMH and vlPAG versus the increase in the PFX following coactivation of GALR2 and NPYY1R might be a consequence of the reduced input to these regions from the pathway of the medial subnucleus of the central amygdaloid nucleus. Importantly, behavioral effects and cFos expression were restored by the GALR2 antagonist, demonstrating the requirement of GALR2 in these processes. In line with these results, the formation of GALR2/NPYY1R heteroreceptor complexes required GALR2, as knockdown of GALR2 reduced the density of complexes in ITCp-dl.

Our behavioral results demonstrated that GAL and the NPYY1 agonist coinjection increased rearing behavior in the open field, elevated plus maze and the light-dark box. Rearing has been considered an exploratory behavior, a mean of sampling or scanning the environment or a marker of environmental novelty (Fernandez Espejo, 1997; Ho et al., 2002; McGregor et al., 2004). However, like other ethological parameters, rearing has been used as an indicator of anxiety in the open field (Lamprea et al., 2008), the elevated plus-maze and the light-dark box (Lepicard et al., 2000). In some studies, increased rearing seems to concord with decreased anxiety (Camara et al., 2015; Choi et al., 2015). Furthermore, reductions in the number of rearings seems to indicate heightened anxiety responses (Rodgers et al., 2002; Shepard and Myers, 2008). Thus, the increase on rearing in all three tasks may be attributed to a consequence of anxiolytic-like response after GAL and the NPYY1 agonist coinjection.

In the elevated plus-maze decreased SAP have been observed after coinjection of GAL and the NPYY1 agonist. SAP is considered to reflect a risk assessment behavior (Blanchard et al., 2011), that occurs when the animal is undergoing risk-assessment, specifically due to an internal exploratory-anxiety conflict. During exploratory-anxiety conflict situations, SAP has been shown a valid measure of anxiety since anxiolytic drugs successfully reduced SAP in the elevated plus-maze (Grewal et al., 1997; Rey et al., 2012). In fact, SAP has been found to be more sensitive to the effects of classical and atypical anxiolytics than traditional spatiotemporal indices in the elevated plus maze (Griebel et al., 1997). Otherwise, high levels of anxiety are thought to be associated with an increased number of SAP (Shepherd et al., 1994; Rodgers, 1997).

Moreover, GAL and NPYY1 agonist coinjection increased head-dipping behavior, looking down over the edges of the elevated plus-maze. The number of head dips is considered an index of anxiety, and its increase is associated with a decrease in anxiety (Cruz et al., 1994). Thus, treatment with alprazolam and diazepam were reported to induce anxiolytic responses in spatiotemporal parameters and increase the frequency of head-dipping in the elevated plus-maze (Dalvi and Rodgers, 1999; Sorregotti et al., 2013). In addition, GAL and NPYY1 agonist coadministration increased latency to enter the dark compartment in the light-dark box. This effect has been related to an anxiolytic response (Aslam and Sultana, 2016) and supports the entire behavioral profile described after GAL and NPYY1 agonist coinjection. Thus, from the above discussion it seems reliable to integrate the present ethological results with our previous (Narvaez et al., 2015) spatiotemporal findings to give each other an enhanced ethological validity.

Importantly, our results demonstrate the relevance of GALR2 as an enhancer of the NPYY1R agonist activity within the GALR2/NPYY1R interaction, since the GALR2 antagonist M871 counteracted the responses observed, as previously described with spatiotemporal parameters (Narvaez et al., 2015). The GALR2 antagonist M871 alone lacked effects in the dose of 3 nmol in all the parameters analyzed, in agreement with the absence of effects described previously (Narvaez et al., 2015, 2016). These behavioral effects observed in the three tasks were independent of the motor activity, since neither GAL and NPYY1 agonist nor their coadministration has shown locomotor alterations, according to previous results (Narvaez et al., 2015).

ITCp neurons are polarized in such a manner that GABAergic cells from dorsolateral located islands inhibit GABA neurons from clusters lying in a more ventromedial position, which regulate central amygdala output neurons producing anxiety (Royer et al., 2000; Palomares-Castillo et al., 2012). Intriguingly, GAL alone increased c-Fos IR in the ITCp-dl, but no effects on behavior were found. Due to the nature of amygdala microcircuits, it is possible that the c-Fos effect observed after GAL administration was not sufficient to induce behavioral responses in unconditioned paradigms, in accordance with previous results (Narvaez et al., 2015). Moreover, galaninergic modulation of fear conditioning warrants further investigation since expression of the immediate-early gene Zif268 increased during fear recall in ITCp-dl (Duvarci and Pare, 2014).

The enhanced reduction of c-Fos profiles observed in the ITCp-dl after GAL and NPYY1R agonist coinjection was induced by GALR2 since it was blocked by the GALR2 antagonist M871. This inhibitory effect induced by the combined agonist treatment may involve an increased inhibition of AC-PKA-CREB pathway in the ITCp-dl GABA cells through enhanced Gi/o signaling of the NPYY1R together with a switching of GALR2 linked Gq signaling to Gi/o mediated signaling taking place in GALR2/NPYY1R heteroreceptor complexes (Narvaez et al., 2015). Decreased c-Fos after GALR2/NPYY1R coactivation was observed specifically in ITCp-dl, and not in the ITCp-vm. This inhibition of ITCp-dl can lead to disinhibition of ITCp-vm projecting to and inhibiting the medial efferent subdivision of the central amygdaloid nucleus, based on current knowledge of amygdala microcircuits (Pare and Duvarci, 2012). This may result in a reduction of the efferent anxiogenic outflow from the amygdala to the hypothalamus and PAG and may give the mechanism at the brain circuit level for the anxiolytic behavioral pattern observed upon cotreatment with GAL and the NPYY1R agonist.

Ventromedial hypothalamic (VMH) nucleus receives strong inputs from the amygdala and is an important part of the medial hypothalamic defensive system, involved in integrating innate defensive responses to environmental threats (Canteras et al., 1997, Canteras, 2002). The c-Fos mapping studies have shown that exposure to different types of threat recruits independent brain circuits within the medial hypothalamus, specifically in the VMH (Silva et al., 2013). Particularly, increased c-Fos IR in the VMH was related to an increase in stretch postures, freezing and a decrease in locomotion (Silva et al., 2013). Also, previous reports indicated that optogenetic activation of VMH cells elicited immobility and defensive behaviors (Lin et al., 2011; Wang et al., 2015). Indeed, we observed a decreased c-Fos IR in the VMH nucleus after GAL and the NPYY1R agonist coinjection, specifically mediated by GALR2 since it was blocked with the GALR2 antagonist M871. This decreased c-Fos IR may be related with the behavioral patterns observed after GAL/NPYY1R agonist coinjection, mimicking the pharmacogenetic inactivation of the VMH that reduces freezing and defensive responses to a natural predator (Silva et al., 2013). The medial hypothalamic defensive system is connected with the PFX, also a key hypothalamic site for eliciting integrated defensive behavior, where electrical or chemical stimulation evokes either attack or escape responses (Roeling et al., 1994). In fact, a positive correlation was found between the number of PFX orexin neurons (Calbindin positive) expressing c-Fos and rearing behavior (Li et al., 2011; Bhagwandin et al., 2013). Accordingly, the observed increase in c-Fos IR profiles in the current work in calbindin/orexin neurons within the PFX would be linked to the increased rearing behavior observed after GAL and NPYY1R agonist coinjection.

Periaqueductal gray is a downstream structure shown to be critical for the expression of fear responses involved in motor pattern initiation (Brandao et al., 2008). It receives directs inputs from key forebrain regions involved in regulation of defensive behavior, such as the amygdala (Tovote et al., 2016) or the VMH (Wang et al., 2015). Thus, the decreased of c-Fos IR profiles observed in the vlPAG could participate in the behavioral pattern noticed after NPYY1R agonist treatment or its combined treatment with GAL.

A lack of c-Fos modifications was observed in the PVN. Also, no changes were detected in corticosterone blood levels after GAL and NPYY1 agonist alone or their combined treatment. These results are in agreement with the lack on c-Fos IR modifications in the PVN and corticosterone response observed after GAL (Mitsukawa et al., 2009) or NPY injections (Sajdyk et al., 2008). In fact, several studies suggest a dissociation between the effects of several anxiolytic drugs, such as NPY or diazepam, and the hypothalamic-pituitary axis (HPA) response, probably due to their differential regulation through different pathways (Wilson et al., 2004; Sajdyk et al., 2008). As expected for the anxiolytic effects of the coadministration of GAL and NPY Y1 agonists, a lack of c-Fos modifications was observed in the CeM. Further work by using a combination of electrophysiological and optogenetic approaches, might demonstrate modifications in the firing rate of CeM output neurons (Duvarci and Pare, 2014).

As previously pointed out, we observed the presence of GALR2/NPYY1R heteroreceptor complexes specifically in the ITCp-dl compared with ITCp-vm in control animals. Moreover, in the ITCp-dl a specific and significant increase was demonstrated in these heteroreceptor complexes upon combined treatment with GAL and the NPYY1R agonist. It is worth mentioning, however, that *in situ* PLA presents some limitations, this method only show that two proteins are in close proximity and, therefore, likely directly interact. Proteins could also interact indirectly through an adapter protein. Nevertheless, the functional distance obtained is usually close to the one detected in a FRET assay (Borroto-Escuela et al., 2016). Importantly, the increase in the GALR2/NPYY1R heteroreceptor complexes in the ITCp-dl may produce the specific enhancement of the reduction of c-Fos profiles observed upon combined agonist treatment. Indeed, we have previously described how the existence of GALR2/NPYY1R heteroreceptor complexes was linked to a decrease of c-Fos IR at the cellular level (Narvaez et al., 2015, 2016). The mechanism underlying this c-Fos response could be that the coactivation of the receptor protomers of the GALR2-NPYY1R heteroreceptor complex altered the receptor-receptor interactions leading to a more marked Gi/o-mediated inhibition of the AC-PKA-CREB pathway (Narvaez et al., 2015). The GALR2 activation was essential since the presence of the GALR2 antagonist M871 blocked the effect. Intriguingly, no marked reduction of GALR2/NPYY1R heteroreceptor complexes was detected in GALR2 knockdown rats. This may be due to the fact that GALR2 also takes part in GALR2/GALR2 homodimers, GALR1/GALR2 heterodimers or putative GALR1/GALR2/5HT1A heteroreceptor complexes (Borroto-Escuela et al., 2017). It seems as if that siRNA used for knockdown of GALR2 avoided off-target effects on gene regulation since GALR2 siRNA reduction did not affect GALR1 expression (Millon et al., 2015; Flores-Burgess et al., 2017).

As discussed above, GALR2/NPYY1R heteroreceptor complexes may alter the intercalated GABA neuronal circuits which leads to increased inhibition of the medial subnucleus of the central amygdala with reduction of the anxiogenic outflow. Thus, the GALR2-NPYY1R interaction in the ITCp-dl may exert crucial and discrete effects on the activity of the hypothalamus-PAG axis contributing to the anxiolytic actions observed.

**FIGURE 9:**
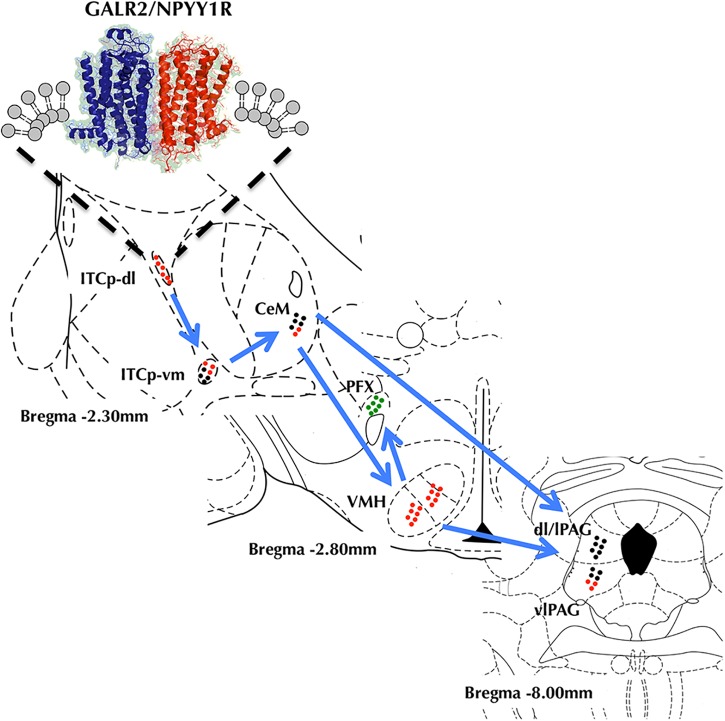
Model diagram showing the relationship and circuit activity modifications on the amygdala-hypothalamus-PAG axis caused by coactivation of GALR2 and NPYY1R from upstream to downstream. GALR2/NPYY1R heteroreceptor complexes would inhibit activity in the dorsolateral cluster of medial paracapsular intercalated nucleus of the amygdala (ITCp-dl) inducing disinhibition of the ventromedial cluster of medial paracapsular intercalated nucleus of the amygdala (ITCp-vm) and reducing medial central amygdala (CeM) output. Thus, whereas ventromedial hypothalamus (VMH) gets inhibited, perifornical región (PFX) it is activated. Within the PAG, the ventrolateral región (vlPAG) gets a reduced activity. Color code (Green dots, Neuronal activation. Red dots, Neuronal inhibition. Black dots, No differences in neuronal activity).

Taken together, we propose based in previous and present results carried out in innate models of anxiety a model for the circuit activity modifications induced by GALR2 and NPYY1R coactivation (**Figure 9**). The current model indicates that coactivation of GALR2 and NPYYR1 increases the GALR2/NPYY1R heteroreceptor complexes in the ITCp-dl associated with enhanced reduction of neuronal activity indicated by reduction of cFos expression. At the network level it appears to reduce the anxiogenic output of the amygdala resulting in anxiolytic-related behaviors. Thus, our results may provide the basis for the development of heterobivalent agonist drugs targeting GALR2/NPYY1R heteromers, especially in the ITCp-dl of the amygdala for the treatment of anxiety.

## Author Contributions

All authors equally contributed to and have approved the final manuscript.

## Conflict of Interest Statement

The authors declare that the research was conducted in the absence of any commercial or financial relationships that could be construed as a potential conflict of interest.
