# Supplementary material for: A Novel Integrative Mechanism in Anxiolytic Behavior Induced by Galanin 2/Neuropeptide Y Y1 Receptor Interactions on Medial Paracapsular Intercalated Amygdala in Rats
Source: Front Cell Neurosci. 2018 May 1;12:119. doi: 10.3389/fncel.2018.00119 (PMC5938606; doi:10.3389/fncel.2018.00119)
Supplement: Supplementary file 1 [file Presentation_1.pdf]

Supplementary Material

A novel integrative mechanism in anxiolytic behavior induced by Galanin 2/Neuropeptide Y Y1 receptor interactions on medial paracapsular intercalated Amygdala in rats

Manuel Narváez*, Dasiel O. Borroto-Escuela, Luis Santín, Carmelo Millón, Belén Gago, Antonio Flores-Burgess, Miguel Angel Barbancho, Miguel Pérez de la Mora, José Angel Narváez, Zaida Díaz-Cabiale & Kjell Fuxe

***Correspondence:**
Manuel Narvaez, M.D.-Ph.D.
Departamento de Fisiología Humana
Instituto de Investigación Biomédica de Málaga
Facultad de Medicina
Universidad de Málaga
Campus de Teatinos s/n. 29071 Málaga, Spain.
Fax: + 34-952131650 Tel.: + 34-952137427
E-mail: mnarvaez@uma.es

Intracerebral cannulations

Rats anesthetized intraperitoneally with Equitesin (3,3ml/Kg) were implanted with a chronic 22- gauge stainless-steel guide cannula (Plastics One In) into the right lateral cerebral ventricle using the following stereotaxic coordinates: +1.4mm lateral, -1mm posterior to the bregma, and 3.6mm below the surface of the skull (Paxinos and Watson, 1986). After surgery, animals were individually housed and allowed recovery for 7 days. This method of cannulation and postsurgical care has been previously standarized (Narvaez et al., 2015;Narvaez et al., 2016)

Intracerebroventricular administration of peptides

Cannulated rats were randomly allocated to different groups. Peptides were freshly prepared, dissolved in aCSF and injected into the right lateral ventricle. The total

volume was 5 µl per injection with an infusion time of 1 min. The GALR2 agonist M1145 (Ki=6.55 nM for GALR2), NPYY1R agonist [Leu^{31},Pro^{34}]NPY (Ki=0.39 nM for NPYY1R) and GALR2 antagonist M871 (Ki=13.1 and 420 nM for GALR2 and GALR1 respectively) were obtained from Tocris Bioscience (Bristol, UK). Experimental groups and the n size of each group is indicated in the different procedures. After the experiments, brains were removed, testing the placement of the cannula for icv injection by cutting the brain in the coronal plane in a Cryostat (HM550, Microm International). These procedures of intracerebroventricular (icv) injections and preparation of artificial cerebrospinal fluid (aCSF) have already been standarized in our laboratory (Narvaez et al., 2015;Narvaez et al., 2016).

c-Fos immunohistochemistry stereological analysis

An Olympus BX51 microscope (Olympus, Denmark) was interfaced with a computer and a colour JVC digital video camera. For stereological analysis, sampling of c-Fos positive cells was performed throughout the medial paracapsular intercalated nuclei (ITCp) of the amygdala, hypothalamic ventromedial nucleus (VMH), paraventricular hypothalamic nucleus (PVN), perifornical region (PFX) and periaqueductal gray matter (PAG) in the rostrocaudal dimension using the optical fractionator. This method combines the optical dissector with a fractionator sampling scheme to exclude volume divergences (Gundersen et al., 1988). Counterstaining with phase contrast and calbindin-immunoreactive cells allowed delineation of different areas in each section (Paxinos and Watson, 1986). Sections were sampled every 150 µm, starting at the ventral part of the ITCp, VMH, PVN, PFX and PAG (approximately 2.20, 2.30, 1.80, 2.70 and 7.50mm posterior to Bregma, respectively). Sampling ended at approximately 3.00, 3.60, 2.20, 3.50 and 8.20 mm posterior to Bregma at the dorsal part of the ITCp, VMH, PVN, PFX and PAG. Numbers of c-Fos IR profiles were quantified in at least five representative 150 µm, evenly spaced sections per animal (4 rats per group). A random set of sampling frames with a known area (α frame) was generated for each section using the C.A.S.T. Grid (Olympus; Albertslund, Denmark). After the objects were counted (ΣQ-) the total number of positive cells were estimated

as: N = ΣQ- x fs x fa x fh (Gundersen et al., 1988), where fs is the numerical fraction of the section used, fa is the areal fraction and fh is the linear fraction of section thickness. The coefficient of error (CE) for each estimation and animal ranged from 0.05 to 0.1. The total CE of each group ranged from 0.07 to 0.08. Counting of labelled neurons was set starting at 5 μm below the surface and focusing through the 20 μm section optical plane. At least five sections of each region were counted per animal and the number of counting frames used was 90-110 per animal. We have used this stereological procedure is previous studies (Diaz-Cabiale et al., 2011;Narvaez et al., 2015;Narvaez et al., 2016).

Corticosterone assay

After open field and elevated plus maze test trunk blood was collected into EDTA-coated tubes on ice (4°C). Blood samples were centrifuged at 4000 relative centrifugal force; plasma was collected and stored at -80°C. Plasma corticosterone was measured with radioimmunoassay kit (MP Biomedicals, Solon, OH; corticosterone #07-120102). Trunk blood was collected between 10:00 and 12:00am for all animals.

Supplementary Figure 1

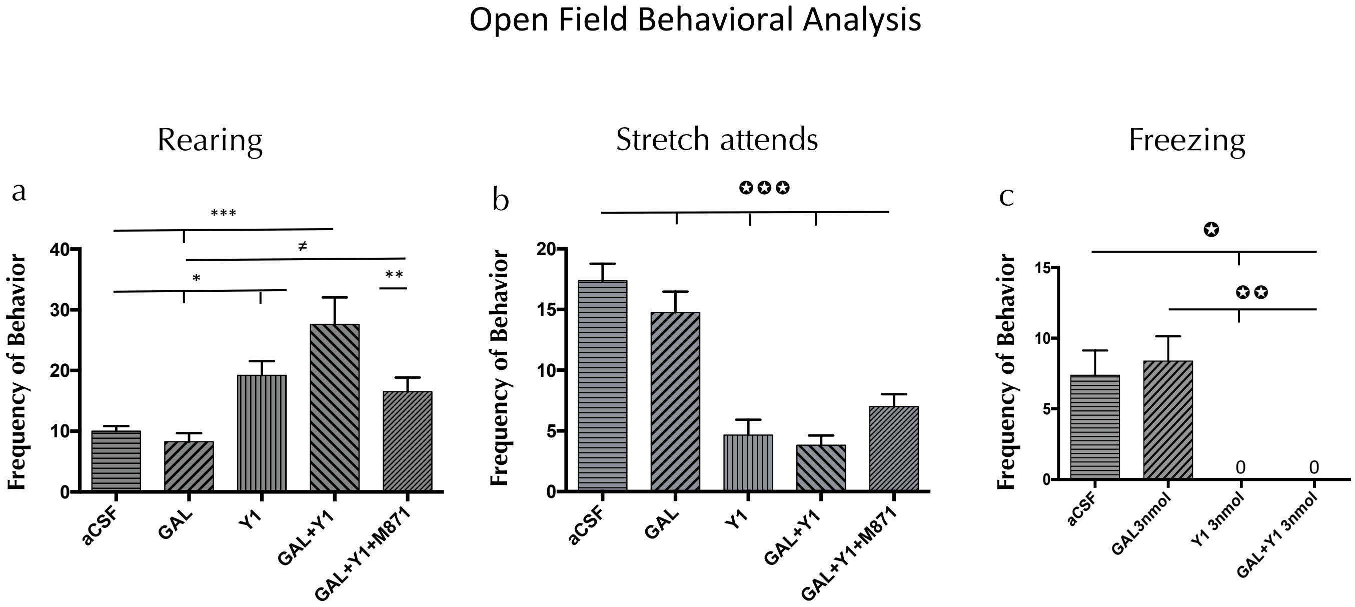

Supplementary Figure 1. Open field behavioral analysis mediated by Galanin and the Neuropeptide Y Y1 receptor agonist. The response induced by GAL on the Y1-mediated ethological behaviors is blocked by the GALR2 antagonist M871. Frequency of rearings **(a)**, strecth attends postures **(b)** and freezing episodes **(c)** in the open field. N=6-8 animals in each test group. Data represents mean ± SEM. Value for GAL+Y1+M871 group on freezing was 0.28±0.28. **P* <0.05; ***P* <0.01; ****P* <0.001; ≠*P* <0.05; ✪*P* <0.05; ✪✪*P* <0.01; ✪✪✪*P* <0.001 according to one-way ANOVA followed by Newman Keuls Multiple Comparison Test. Inter-group comparisons are indicated by the lines above bars. aCSF= Control; GAL = Galanin 3 nmol; Y1 = NPY Y1 receptors agonist [Leu^{31}-Pro^{34}]NPY 3nmol; GAL+Y1 = Coadministration of GAL and Y1; GAL+Y1+M871= Coadministration of GAL, Y1 and GALR2 antagonist M871 3nmol.

Supplementary Figure 2

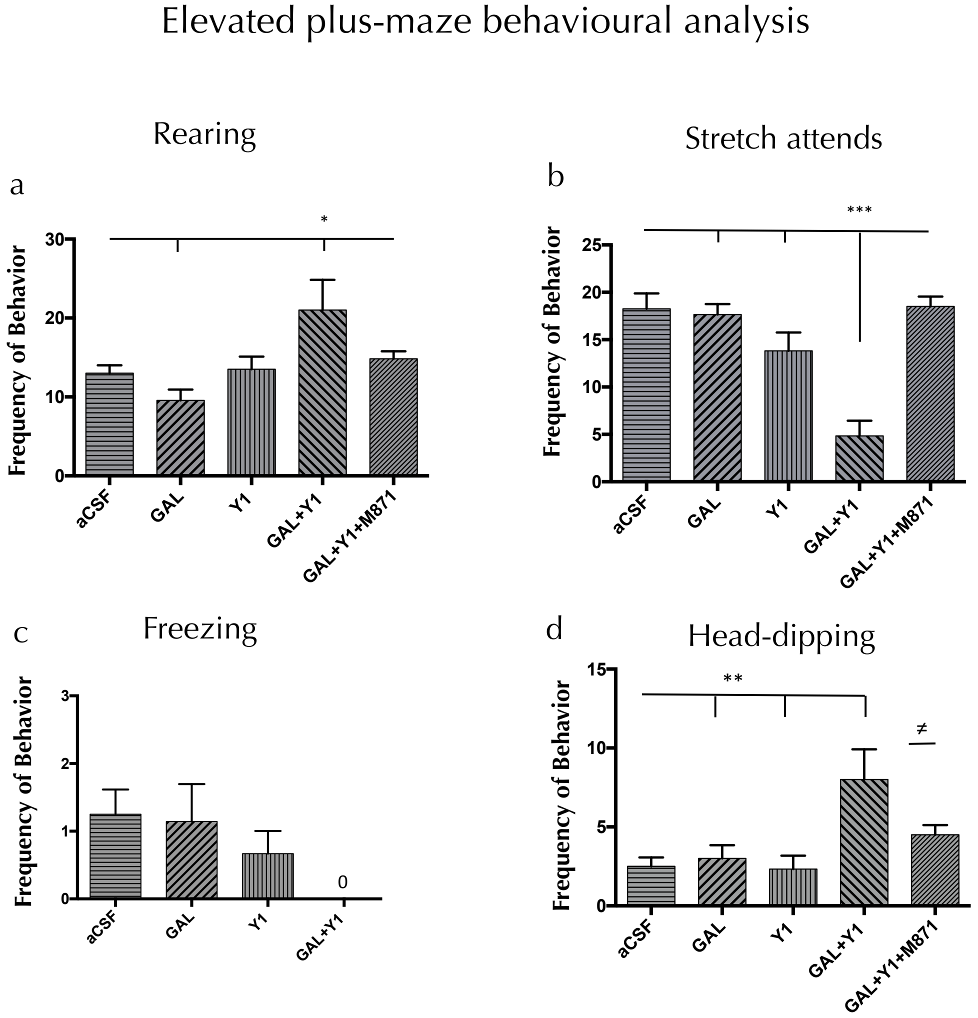

Supplementary Figure 2. Behavioral analysis mediated by Galanin and the Neuropeptide Y Y1 receptor agonist in the elevated plus-maze. The response induced by GAL on the Y1-mediated ethological behaviors is blocked by the GALR2 antagonist M871. Frequency on rearings **(a)**, strecth attends postures **(b),** freezing **(c)** and head-dipping **(d)** in the elevated plus-maze. N=6-8 animals in each test group. Data represents mean ± SEM. Value for GAL+Y1+M871 group on freezing was 0. **P* <0.05; ***P* <0.01; ****P* <0.001; ≠*P*<0.05 according to one-way ANOVA followed by Newman Keuls Multiple Comparison Test. Inter-group comparisons are indicated by the lines above bars. aCSF= Control; GAL = Galanin 3 nmol; Y1 = NPY Y1 receptors agonist [Leu^{31}-Pro^{34}]NPY 3nmol; GAL+Y1 =

Coadministration of GAL and Y1; GAL+Y1+M871= Coadministration of GAL, Y1 and GALR2 antagonist M871 3nmol.

Supplementary Figure 3

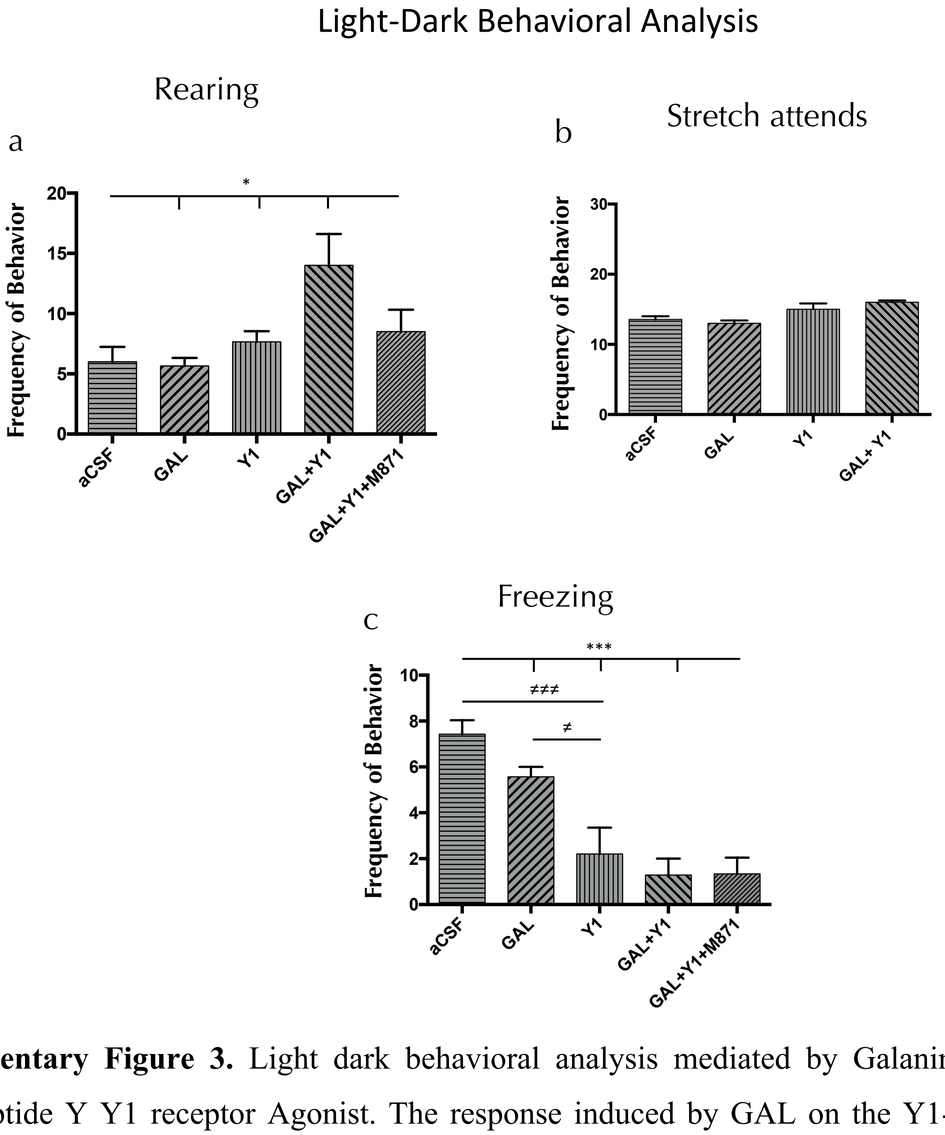

Supplementary Figure 3. Light dark behavioral analysis mediated by Galanin and the Neuropeptide Y Y1 receptor Agonist. The response induced by GAL on the Y1-mediated ethological behaviors is blocked by the GALR2 antagonist M871. Frequency on rearings **(a)**, strecth attends postures **(b)** and freezing episodes **(c)** in the Light dark box. N=6-8 animals in each group. Data represents mean ± SEM. Value for GAL+Y1+M871 group on Stretch attends was 14.4±0.73. *P <0.05; ***P <0.001; ≠P <0.05; ≠≠≠P <0.001; according

to one-way ANOVA followed by Newman Keuls Multiple Comparison Test. Inter-group comparisons are indicated by the lines above bars. aCSF= Control; GAL = Galanin 3 nmol; Y1 = NPY Y1 receptors agonist [Leu^{31}-Pro^{34}]NPY 3nmol; GAL+Y1 = Coadministration of GAL and Y1; GAL+Y1+M871= Coadministration of GAL, Y1 and GALR2 antagonist M871 3nmol.

Supplementary Figure 4

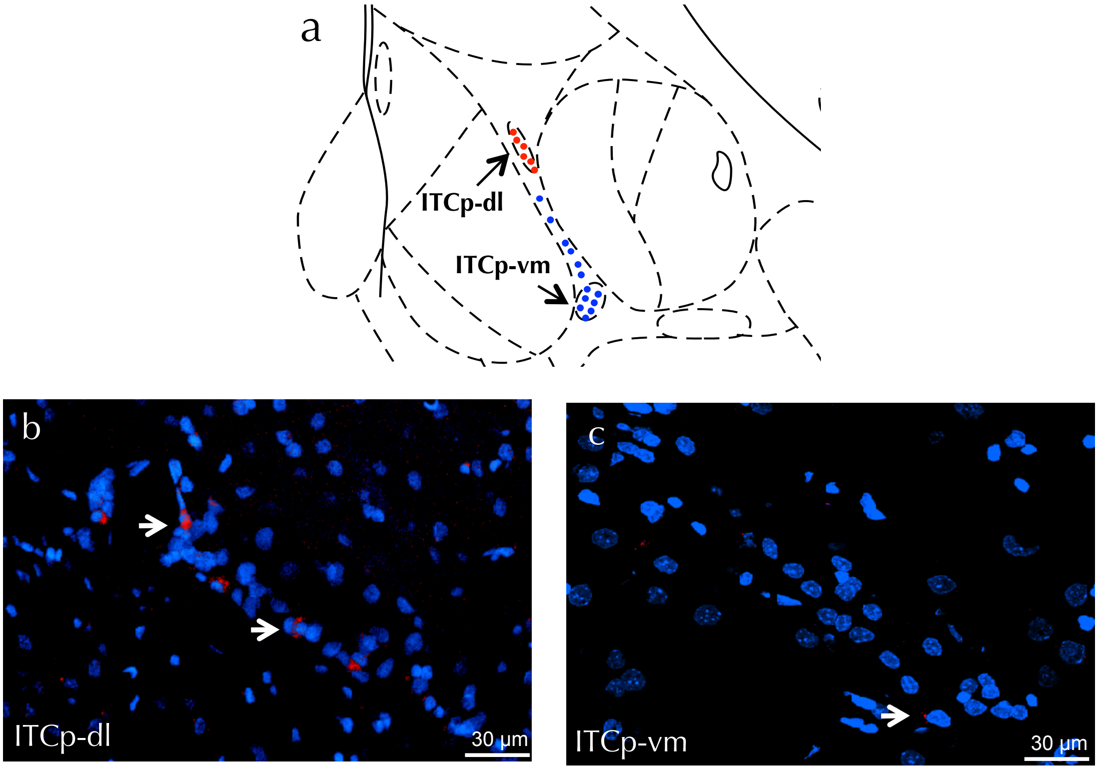

Supplementary Figure 4. Detection of GALR2/NPYY1R heteroreceptor complexes with *in situ* proximity ligation assay (PLA) in the dorsolateral cluster of medial paracapsular intercalated nucleus of the amygdala (ITCp-dl). **(a-c)** Diagram and representative microphotographs showing the presence of positive PLA signals (red circles) in the ITCp-dl **(b)** and lack of specific signals (blue circles) in the ventromedial cluster (ITCp-vm) **(c).** Bregma: -2.3mm according to the sterotaxic atlas of Paxinos and Watson (1986). GALR2/NPYY1R heteroreceptor complexes are shown as red PLA blobs (clusters) found in high densities per cell in a large number of nerve cells using confocal laser microscopy, White arrows point to PLA clusters. The nuclei are shown in blue by DAPI.

Supplementary Table 1

c-Fos-IR	aCSF	GAL	Y1	GAL+Y1
PVN	51 ± 5.4	59 ± 6.7	57 ± 10.5	46 ± 4
CeM	20 ± 10	36 ± 4	16 ± 6.5	12.8 ± 5.9

Supplementary Table 1. Number of total c-Fos IR profiles expression after Galanin (GAL) and NPY Y1 receptor (NPYY1R) agonist, alone or in combination in the paraventricular nucleus (PVN) of the hypothalamus and the medial part of the central (CeM) Amygdala. Data, expressed as mean ± SEM, show no differences between groups according to one-way ANOVA. aCSF= Control; GAL = Galanin 3 nmol; Y1 = NPY Y1 receptors agonist [Leu^{31}-Pro^{34}]NPY 3nmol; GAL+Y1 = Coadministration of GAL and Y1.

Supplementary Table 2

Corticosterone levels (ng/ml)	aCSF	GAL	Y1	GAL+Y1
OF	356.3±74	400±52	501±59	474±48
EPM	455±15	476±52	383.4±62	441.9±23

Supplementary Table 2. Corticosterone levels found in rats injected with GAL and NPYY1R agonist alone or in combination after open field (OF) o elevated plus maze (EPM). Data, expressed as mean ± SEM, show no differences between groups on corticosterone levels according to one-way ANOVA. aCSF= Control; GAL = Galanin 3 nmol; Y1 = NPY Y1 receptors agonist [Leu^{31}-Pro^{34}]NPY 3nmol; GAL+Y1 = Coadministration of GAL and Y1.

Supplementary Table 3

Distance (cm)<br>Speed (cm/s)	aCSF	GAL	Y1	GAL+Y1
OF	3139±494<br>9.72±1.1	2893±144<br>9.61±0.49	3041±199<br>10.1±0.66	3352±143<br>9.19±0.27
EPM	1086±38.6<br>3.9±0.17	1189±139<br>3.95±0.96	1142±43.7<br>3.93±0.29	1145±33<br>3.92±0.21
LD	822±35.4<br>2.7±0.14	854.2±92.7<br>2.8±0.4	845±32.3<br>2.9±0.32	881±29.8<br>2.85±0.16

Supplementary Table 3. Analysis of locomotor activity parameters in the open field (OF), elevated plus maze (EPM) and light-dark box (LD). Data shown as mean ± SEM. No differences were found according to one-way ANOVA. aCSF= Control; GAL = Galanin 3 nmol; Y1 = NPY Y1 receptors agonist [Leu^{31}-Pro^{34}]NPY 3nmol; GAL+Y1 = Coadministration of GAL and Y1.

References

Diaz-Cabiale, Z., Parrado, C., Narvaez, M., Puigcerver, A., Millon, C., Santin, L., Fuxe, K., and Narvaez, J.A. (2011). Galanin receptor/Neuropeptide Y receptor interactions in the dorsal raphe nucleus of the rat. *Neuropharmacology* 61**,** 80-86.

Gundersen, H.J., Bagger, P., Bendtsen, T.F., Evans, S.M., Korbo, L., Marcussen, N., Moller, A., Nielsen, K., Nyengaard, J.R., Pakkenberg, B., and Et Al. (1988). The new stereological tools: disector, fractionator, nucleator and point sampled intercepts and their use in pathological research and diagnosis. *APMIS* 96**,** 857-881.

Narvaez, M., Borroto-Escuela, D.O., Millon, C., Gago, B., Flores-Burgess, A., Santin, L., Fuxe, K., Narvaez, J.A., and Diaz-Cabiale, Z. (2016). Galanin receptor 2-neuropeptide Y Y1 receptor interactions in the dentate gyrus are related with antidepressant-like effects. *Brain Struct Funct* 221**,** 4129-4139.

Narvaez, M., Millon, C., Borroto-Escuela, D., Flores-Burgess, A., Santin, L., Parrado, C., Gago, B., Puigcerver, A., Fuxe, K., Narvaez, J.A., and Diaz-Cabiale, Z. (2015). Galanin receptor 2-neuropeptide Y Y1 receptor interactions in the amygdala lead to increased anxiolytic actions. *Brain Struct Funct* 220**,** 2289-2301.
Paxinos, G. and Watson, C. (1986) The Rat Brain in Stereotaxical Coordinates. Second edition. Academic Press, San Diego.
